# Serological profile of naïve patients affected by the first sars-cov-2 variant: A prospective study

**DOI:** 10.1371/journal.pone.0344308

**Published:** 2026-03-09

**Authors:** Wafa Dhouib, Meriem Kacem, Oumayma belghayeb, Meriem Oumaima Beji, Cyrine Bennasrallah, Ameni Maatouk, Imen Zemni, Hela Abroug, Ines bouanene, Haythem Sriha, Maha Mastouri, Mourad ghali, Asma Sriha Belguith, Manel Ben Fredj

**Affiliations:** 1 Department of Epidemiology and Preventive Medicine, Fattouma Bourguiba University Hospital, Monastir, Tunisia; 2 Department of Epidemiology, Faculty of Medicine of Monastir, University of Monastir, Monastir, Tunisia; 3 Technology and Medical Imaging Research Laboratory - LTIM - LR12ES06, University of Monastir, Monastir, Tunisia; 4 Department of Family Medicine, Faculty of Medicine of Monastir, Monastir, Tunisia; 5 Department of pediatric surgery, Faculty of Medicine of Monastir, Monastir, Tunisia; 6 Laboratory of Microbiology, Fattouma Bourguiba University Hospital of Monastir/ University of Monastir/ Faculty of Medicine of Monastir, Monastir, Tunisia; 7 Department of Immunology, Fattouma Bourguiba University Hospital, Monastir, Tunisia; UniCamillus: Saint Camillus International University of Health and Medical Sciences, ITALY

## Abstract

**Background:**

Understanding post-infection immunity with the first SARS-CoV-2 variant may provide valuable insights into the duration and effectiveness of the humoral immune response. This study aims to characterize the serological profile of naïve individuals infected with the first SARS-CoV-2 variant.

**Methods:**

A prospective study with repeated measures was conducted in Tunisia, from March to October 2020, during the first wave of COVID-19. Adults confirmed with confirmed COVID-19 were monitored during the first wave of the pandemic. ELISA blood tests were conducted at multiple intervals: day 7, day 14, and at 1, 2, 3, 4, and 6 months post-infection.

**Results:**

173 serum samples were collected from immunologically naïve individuals infected with the first circulating SARS-CoV-2 variant, ranging from 7 days to 6 months post-RT-PCR confirmation. The study revealed a robust humoral immune response in most participants, with 94.1% testing positive for IgM anti-N, 88.2% for IgM anti-S, 98% for IgG anti-N, and 100% for IgG anti-S antibodies. Anti-N IgM antibodies peaked at days 14 and 30 with high positive values (>0.260), while anti-S IgM antibodies showed elevated levels (>0.990) at days 7 and 14. For IgG, anti-N antibodies reached their highest levels (>0.810) at month 4, while anti-S IgG antibodies maintained high positive values (>0.490) at days 7 and 14, and remained elevated at months 4 and 6. No significant differences in antibody levels were observed based on gender, age, comorbidities, or symptoms presence.

**Conclusion:**

A typical adaptive immune response was observed in naïve individuals infected with the initial SARS-CoV-2 variant, showing typical IgM and IgG antibody production from day 7 to month 6. We specifically investigated immunologically naïve individuals infected with the first circulating SARS-CoV-2 variant, from the earliest stage of infection, a context that is no longer reproducible.

## 1. Introduction

On March 11, 2020, the WHO declared the COVID-19 pandemic. The first variant was named using the Greek alphabet (Alpha). The natural humoral and cellular immune responses were the primary lines of defense [1–4]. Case studies on SARS-CoV-2 reinfections indicate that the natural immune response may not offer lasting protection [5,6]. This realization quickly accelerated the pursuit of vaccines capable of providing durable immunity to curb the pandemic. However, compounding the challenge was the repeated emergence of new variants [7–9]. The ELISA test was the most effective for antibody detection. IgM antibodies are the first produced by the immune system following B cell activation, playing a key role in the early control of infection. IgG antibodies, produced by serum cells, contribute to both immediate and long-term immunity against the triggering antigen. Anti-S antibodies target the Spike protein, particularly the S1 subunit with the receptor-binding domain (RBD), which is essential for virus neutralization. Anti-N antibodies target the nucleocapsid protein, responsible for RNA genome binding, replication, and the formation of virus-like particles, thus indicating past infection. Over 90% of neutralizing antibodies target the spike and nucleocapsid proteins in infected individuals. Analyzing serological profiles early in the pandemic should have provided insights into the immune response to COVID-19 [7,10–14]. Global studies have shown disparities in COVID-19 morbidity and mortality across different ethnicities and populations. Tunisia was heavily impacted, with a notably high fatality rate [15,16]. From the beginning of the pandemic, our research lab received approval from the Ministry of Health to conduct a pilot survey on natural immunity to SARS-CoV-2, as no research had yet detailed the humoral response to the original variant before the introduction of vaccines.

Objective: We aimed to describe the serological profile of SARS-CoV-2-naïve individuals infected with the first identified variant.

## 2. Methods

### 2.1. Study design

We conducted a prospective observational study with repeated measures among immunologically naïve individuals infected with the first circulating SARS-CoV-2 variant who were isolated in the national isolation centers, between March 15 and October 20, 2020, in Monastir (Tunisia).

### 2.2. Setting

In Tunisia, the first COVID-19 wave lasted from March 2 to June 18, 2020. Various measures were implemented to limit the spread of the disease. Among these interventions, the one that provided us with the opportunity to conduct this study was the quarantine of confirmed cases in national isolation centers, in Monastir hotels [17]. In addition to on-site teams, the Department of Preventive Medicine and Epidemiology at Monastir University Hospital provided daily telephone follow-up and performed RT-PCR testing. Isolation was lifted after two consecutive negative RT-PCR results, 24 hours apart, with testing conducted every seven days.

Following the first wave, easing of restrictions led to a resurgence, and a “stop-and-go” approach was implemented from June to October 2020. The National vaccination strategy was launched in March 2021 under the COVAX initiative [18,19]. At the pandemic’s outset, the Federated Research Project (FRP) was initiated in Tunisia, with the preventive medicine, microbiology, and physical medicine research laboratory of the Faculty of Medicine of Monastir (LR12ES06) submitting a project to study the serological profile of patients infected with the first SARS-CoV-2 variant.

### 2.3. Study population

Inclusion criteria: Adults with confirmed COVID-19 via RT-PCR who provided informed consent were eligible for inclusion.

Non-inclusion criteria: Patients under 18 years of age, as well as those in critical condition and unable to provide consent, were not included.

### 2.4. Data collection

At the national isolation centers, trained healthcare professionals from the Preventive Medicine Department (PMD) thoroughly explained the study protocol to participants. Upon obtaining informed consent, they collected sociodemographic variables (age, gender, phone number, home region, medical history, medications) and COVID-19-related data (symptoms, type and onset date, infection confirmation date, recovery date, hospitalization, treatment). A blood sample was then collected in dry tubes and sent to the virology lab for centrifugation and storage at −80°C. Once enough samples were gathered, ELISA tests were conducted.

**Fig 1:**
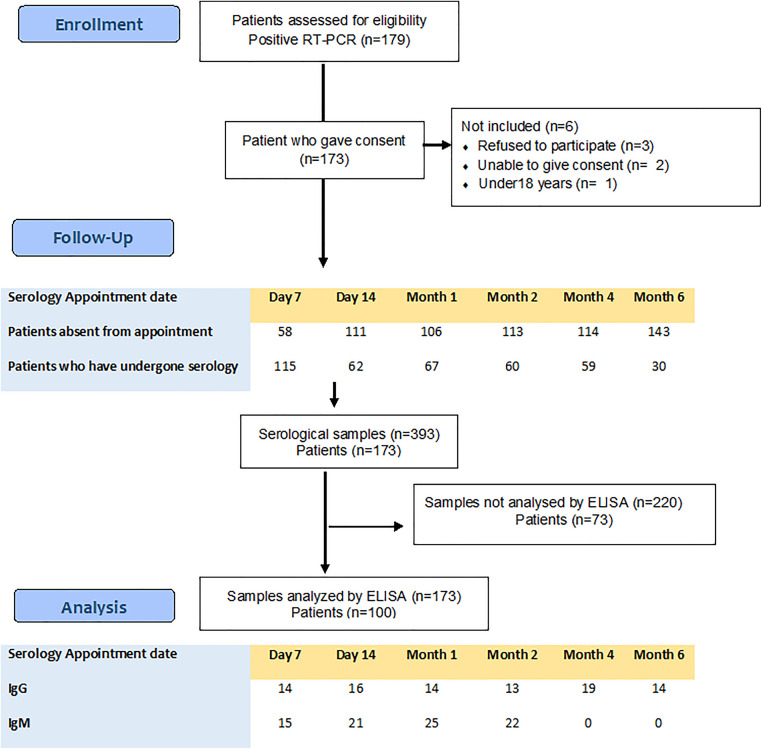
Patient enrollment, follow-up, and serological study analysis flowchart.

To ensure confidentiality, each sample was assigned a unique, coded identification number. The same blood sampling procedures were performed prospectively at different time points: on the date of first confirmation, day 7, day 14, month 1, month 2, month 3, month 4, and month 6. Each patient was free to withdraw from the study at any time. Patients who missed prior appointments could continue with the protocol on subsequent dates. The results were provided to all participants. The Federated Research Project (FRPCOV19-D2P1) selection was made on March 15, 2020, with a detailed protocol approved on April 30, 2020. Recruitment and serum sampling were initiated at isolation centers after notifying regional directors and center supervisors. Patients were included at days 7, 14, and months 1 or 2 post-infection, with six serum samples planned per patient. Adherence was limited due to late inclusion, isolation-related discomfort, confinement, or premature discharge, and a total of 393 serum samples were collected and stored frozen. Project funding became effective on October 30, 2020, but covered only half the requested amount, permitting purchase of two kits per protein type; subsequent funding was not released due to economic difficulties. To optimize limited resources, a random selection of samples from each time point (days 7, 14, months 1–6) was analyzed to describe longitudinal serological kinetics. While full-cohort analysis would increase precision, the selected sample size was sufficient given the novelty of the findings (Fig 1).

### 2.5. Measurements

ELISA (enzyme-linked immunosorbent assay) is a widely used immunological test for detecting antibodies and proteins in biological samples. This technique visualizes antigen–antibody interactions through a colored enzyme marker.

A venous blood sample was collected in dry tubes and transported to the virology laboratory, where samples were centrifuged at 3000 rpm for 15 minutes to obtain serum, which was aliquoted and stored at −80°C until analysis. Serological analyses were performed using a commercial ELISA kit (Proteintech™, KE30005-96T) for the detection of anti-SARS-CoV-2 IgG and IgM antibodies directed against the S1 and N proteins.

According to the manufacturer’s datasheet, the assay demonstrates high analytical performance, with reported high analytical sensitivity and specificity values and intra- and inter-assay coefficients of variation below 10%.

Briefly, SARS-CoV-2 antigens are pre-coated onto microplate wells. Patient serum samples were added, allowing specific antibodies to bind to immobilized antigens. After washing, a horseradish peroxidase-conjugated secondary antibody was applied according to the kit protocol.

Signal development was achieved using 3,3’,5,5’-tetramethylbenzidine (TMB) substrate, and the reaction was stopped with sulfuric acid. Optical density was measured at 450 nm within the time window recommended by the manufacturer.

Optical density cut-off values for positivity were defined according to the manufacturer’s recommendations as:

- 0.078 for IgG anti-N,
- 0.0861 for IgG anti-S,
- 0.022 for IgM anti-N,
- 0.041 for IgM anti-S.

Antibody levels were categorized as follows:

Interpretation of antibody levels response (IgG and IgM) in post-infection with the first SARS-CoV-2 Variant.

**Table:** 

Antibody	weak positive	medium positive	high positive
IgG anti-N	0.078–0.449	0.45–0.810	>0.810
IgG anti-S	0.0861–0.259	0.260–0.490	>0.490
IgM anti-N	0.022–0.139	0.140–0.260	>0.260
IgM anti-S	0.041–0.229	0.230–0.990	>0.990

We employed a binary age categorization (≤40 years vs. > 40 years), as 40 years represents a critical threshold for the emergence of comorbidities that can modulate immune response outcomes [20,21].

### 2.6 Statistical analysis

Data entry and statistical analysis were carried out using the IBM SPSS Statistics 22 software package. Quantitative data were expressed as means and medians, while qualitative data were presented as percentages. The Chi-square test and Fisher’s exact test were appropriately used for qualitative variables and percentage comparisons.

For non-parametric tests, the Mann-Whitney test was employed to compare the median of quantitative variables. Values were considered significant when p ≤ 0.05.

### 2.7 Ethical considerations

Written informed consent was obtained from each individual before enrollment in this survey. This study was approved by the ethics committee of the Faculty of Medicine in Monastir under the reference number IORG 0009738N57/OMB 0990−0279.

## 3 Results

### 3.1 Description of the study population

A total of 100 participants were included, with a mean age of 36 years (SD: 1) and a male-to-female ratio of 1.53. Comorbidities were present in 23% of participants, and 52% were asymptomatic (Table 1).

**Table 1:** Demographic and clinical profiles of study population (Tunisia 2020).

Characteristics	N (%)
Overall	100(100)
Age (years) mean(SD)	36 (13)
Sexe:	
Female:	39(39)
Male:	61(61)
Symptoms:	
Symptomatic	48(48)
Asymptomatic	52(52)
Smoking:	
Smokers	22(22)
Non-smokers	78(78)
Personal medical history:	
No comorbidities	77(77)
One or more comorbidities	23(23)

SD:Standard Deviation.

### 3.2. Kinetics of the humoral response

**Fig 2:**
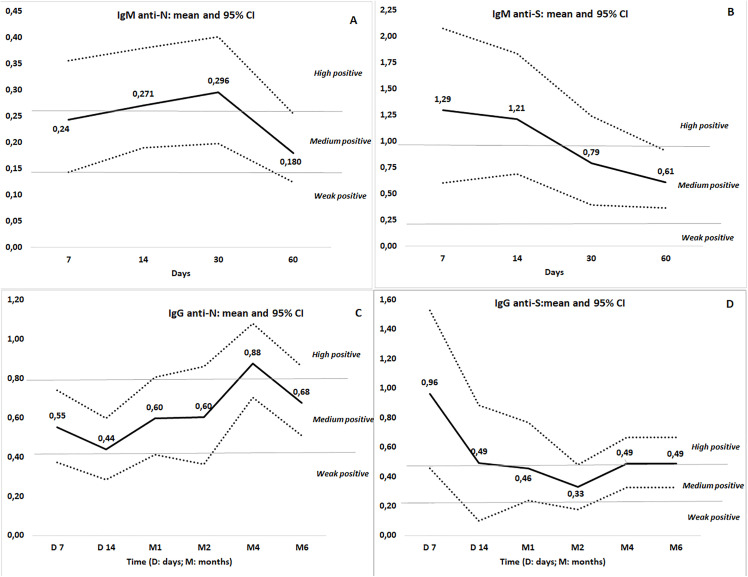
Temporal Evolution of Antibody Responses (IgM and IgG) Against Nucleocapsid (N) and Spike (S) Proteins in Individuals Over Time: Mean and 95% Confidence Intervals.

We analyzed 173 serum samples collected longitudinally. IgM antibodies were detected in most patients (94.1% anti-N, 88.2% anti-S), peaking at days 14–30 for anti-N (>0.260) and days 7–14 for anti-S (>0.990). IgG antibodies were nearly universal (98% anti-N, 100% anti-S), with anti-N peaking at month 4 (>0.810) and anti-S maintaining high levels from days 7–14 through months 4–6 (>0.490) (Fig 2).

### 3.3. Associated factors to serological values

Across the study period (day 7 to month 6), IgG and IgM levels did not differ significantly by sex (Table 2), age (Table 3), comorbidities, or symptom status. Exceptions included: at day 7, symptomatic patients had higher IgG anti-N levels than asymptomatic patients (0.85 [95% CI: 0.55–1.20] vs. 0.43 [95% CI: 0.25–0.63]; p = 0.045). At month 4, symptomatic patients had higher IgG anti-S levels (0.66 [95% CI: 0.40–0.93] vs. 0.29 [95% CI: 0.18–0.40]; p = 0.047) (Table 4).

**Table 2:** Serum antibody levels (IgG and IgM) in male and female subjects over time post- infection with the first SARS-CoV-2 variant.

		All			Male			female		
		Mean	95% CI		Mean	95% CI		Mean	95% CI	
Day 7 (nIgG/nIgM: 14/15)	IgG antiN	0.55	0.37	0.74	0.51	0.31	0.71	0.63	0.22	1.05
	IgG antiS	0.96	0.45	1.53	0.60	0.13	1.06	1.79	0.52	3.05
	IgM antiN	0.24	0.14	0.36	0.20	0.07	0.32	0.33	0.11	0.55
	IgM antiS	1.29	0.60	2.08	1.37	0.27	2.46	1.18	0.01	2.35
Day 14 (nIgG/nIgM: 16/21)	IgG antiN	0.44	0.28	0.60	0.41	0.21	0.61	0.60	0.32	0.87
	IgG antiS	0.49	0.10	0.89	0.58	0.07	1.08	0.21	0.02	0.41
	IgM antiN	0.27	0.190	0.38	0.23	0.17	0.30	0.58	0.00	1.27
	IgM antiS	1.21	0.69	1.84	1.33	0.64	2.02	0.64	0.01	1.27
Month 1 (nIgG/nIgM: 14/25)	IgG antiN	0.60	0.41	0.81	0.44	0.24	0.64	0.77	0.44	1.11
	IgG antiS	0.46	0.24	0.77	0.52	0.17	0.87	0.25	0.09	0.40
	IgM antiN	0.30	0.198	0.40	0.27	0.18	0.37	0.41	0.00	0.92
	IgM antiS	0.79	0.39	1.24	0.97	0.45	1.50	0.16	0.03	0.29
Month 2 (nIgG/nIgM: 13/22)	IgG antiN	0.60	0.36	0.86	0.61	0.27	0.94	0.59	0.15	1.03
	IgG antiS	0.33	0.17	0.48	0.33	0.11	0.56	0.32	0.30	0.34
	IgM antiN	0.18	0.124	0.26	0.14	0.09	0.18	0.24	0.09	0.39
	IgM antiS	0.61	0.36	0.91	0.79	0.36	1.22	0.35	0.07	0.63
Month 4 IgG: n = 19	IgG antiN	0.88	0.70	1.08	0.88	0.40	1.36	0.87	0.72	1.03
	IgG antiS	0.49	0.33	0.68	0.54	0.20	0.88	0.45	0.23	0.66
Month 6 IgG: n = 14	IgG antiN	0.68	0.51	0.86	0.77	0.46	1.09	0.60	0.40	0.80
	IgG antiS	0.49	0.35	0.61	0.60	0.35	0.84	0.38	0.24	0.52

nIgG/ nIgM: number of serum samples tested for IgG and IgM, respectively

**Table 3:** Serum antibody response (IgG and IgM) in different age groups (≤40 and >40 years) post- infection with the first SARS-CoV-2 variant.

		Age ≤ 40			Age > 40		
		Serum Antibody Response	95% CI		Serum Antibody Response	95% CI	
Day 7 (nIgG/nIgM: 14/15)	IgG antiN	0.47	0.36	0.69	0.86	0.86	0.86
	IgG antiS	1.02	0.69	1.69	0.41	0.41	0.41
	IgM antiN	0.26	0.14	0.50	0.21	0.14	0.34
	IgM antiS	1.47	0.76	2.86	0.83	0.41	1.66
Day 14 (nIgG/nIgM: 16/21)	IgG antiN	0.44	0.34	0.64	0.47	0.24	0.90
	IgG antiS	0.56	0.23	1.19	0.44	0.28	0.75
	IgM antiN	0.23	0.19	0.31	0.33	0.21	0.59
	IgM antiS	1.12	0.77	1.80	1.58	0.78	3.15
Month 1 (nIgG/nIgM: 14/25)	IgG antiN	0.54	0.45	0.71	0.53	0.36	0.85
	IgG antiS	0.57	0.31	1.08	0.34	0.31	0.39
	IgM antiN	0.25	0.21	0.34	0.44	0.26	0.77
	IgM antiS	1.03	0.72	1.63	0.38	0.23	0.69
Month 2 (nIgG/nIgM: 13/22)	IgG antiN	0.58	0.39	0.95	0.63	0.42	1.04
	IgG antIS	0.27	0.17	0.47	0.31	0.20	0.54
	IgM antiN	0.22	0.14	0.38	0.14	0.12	0.19
	IgM antiS	0.43	0.26	0.77	0.69	0.46	1.16
Month 4 IgG: n = 19	IgG antiN	0.60	0.53	0.76	1.07	0.94	1.31
	IgG antiS	0.36	0.27	0.55	0.59	0.44	0.89
Month 6 IgG: n = 14	IgG antiN	0.52	0.42	0.72	0.79	0.66	1.04
	IgG antiS	0.38	0.32	0.49	0.55	0.43	0.77

nIgG/ nIgM: number of serum samples tested for IgG and IgM, respectively.

**Table 4:** Serum antibody response (IgG and IgM) in symptomatic and asymptomatic individuals following infection with the first SARS-CoV-2 variant.

		Symptomatic			Non symptomatic			P
		Serum Antibody Response	95% CI		Serum Antibody Response	95% CI		
Day 7 (nIgG/nIgM: 14/15)	IgG antiN	**0.85**	**0.55**	**1.20**	**0.43**	**0.25**	**0.63**	**0.045**
	IgG antiS	1.70	0.38	3.48	0.63	0.31	1.09	0.249
	IgM antiN	0.20	0.04	0.36	0.26	0.13	0.40	0.634
	IgM antiS	1.29	0.00	3.66	1.30	0.54	2.12	0.991
Day 14 (nIgG/nIgM: 16/21)	IgG antiN	0.40	0.16	0.74	0.48	0.24	0.73	0.991
	IgG antiS	0.76	0.17	1.92	0.34	0.17	0.55	0.466
	IgM antiN	0.41	0.18	0.71	0.21	0.15	0.28	0.259
	IgM antiS	1.43	0.41	3.15	1.14	0.61	1.83	0.689
Month 1 (nIgG/nIgM: 14/25)	IgG antiN	0.41	0.26	0.55	0.63	0.34	0.83	0.193
	IgG antiS	0.39	0.21	0.68	0.74	0.11	1.86	0.597
	IgM antiN	0.22	0.15	0.30	0.25	0.13	0.37	0.682
	IgM antiS	0.54	0.12	1.15	1.27	0.52	2.23	0.206
Month 2 (nIgG/nIgM: 13/22)	IgG antiN	0.69	0.40	1.01	0.74	0.49	0.99	0.896
	IgG antiS	0.22	0.11	0.34	0.51	0.31	0.70	0.103
	IgM antiN	0.15	0.13	0.18	0.13	0.03	0.28	0.769
	IgM antiS	0.46	0.27	0.70	1.26	0.09	2.45	0.051
Month 4 IgG: n = 19	IgG antiN	0.96	0.66	1.27	0.72	0.61	0.84	0.260
	IgG antiS	**0.66**	**0.40**	**0.93**	**0.29**	**0.18**	**0.40**	**0.047**
Month 6 IgG: n = 14	IgG antiN	0.66	0.46	0.88	0.70	0.47	0.91	0.893
	IgG antiS	0.48	0.32	0.65	0.44	0.26	0.61	0.829

nIgG/ nIgM: number of serum samples tested for IgG and IgM, respectively.

## 4. Discussion

### 4.1. Main results

A total of 173 serum samples were collected from participants infected with the first SARS-CoV-2 variant, spanning 7 days to 6 months post RT-PCR confirmation. The study demonstrated a robust humoral response in the majority of participants: IgM anti-N 94.1%, IgM anti-S 88.2%, IgG anti-N 98%, and IgG anti-S 100%. IgM antibodies peaked at days 7, 14, and 30, while IgG anti-S remained elevated from days 7–14 and at months 4 and 6, indicating sustained immunity over time. Antibody levels did not significantly differ according to gender, age, comorbidities, or symptom presence, highlighting a consistent immune response across demographic and clinical subgroups.

### 4.2. Interpretation

The study population presented a male predominance and was mainly composed of young adults, consistent with previous observations during the first wave of COVID-19 [22,23]. During the first wave of COVID-19, it was mainly young individuals who went out during the general lockdown to provide for their families’ essential needs, such as food and urgent supplies. Consequently, they were more frequently exposed to potential infection compared to older adults. Stratification by age revealed that individuals over 40 years had a higher frequency of prolonged symptoms (>21 days), supporting the findings of Carlos David et al. [24]. This may be explained by the age-related decline in immune function and the higher prevalence of comorbidities, which together contribute to delayed viral clearance and prolonged inflammatory responses [25].

A classic adaptive immune response was observed in naïve individuals infected with the initial SARS-CoV-2 variant, characterized by sequential IgM and IgG antibody production [14].

Most patients demonstrated seroconversion for both IgM and IgG as early as day 7, suggesting that the adaptive immune response was rapidly mobilized and that antibody seroconversion likely occurred within the first days following viral exposure. This observation is in line with litterature describing early seroconversion kinetics [11,14,26]. In our cohort, IgM peaked on day 14, with all patients testing positive, before declining progressively from the first month onward; 86% remained IgM-positive until month two. These results consistent with those of Andrea Knies et al., who reported peak IgM at day 20 and decline by day 27 [27]. This early and transient IgM response reflects its role as the first line of humoral defense, followed by class switching toward longer-lasting IgG production. Regarding IgG dynamics, antibodies against the nucleocapsid protein (IgG anti-N) were detected in all patients as early as day 7, consistent with their confirmed baseline infection [28]. IgG anti-S antibodies were also observed at day 7, as reported by Antonia Vena et al. in an Italian population [29]. From month 4 to month 6, patients maintained stable IgG levels. This stability highlights the mid-term durability of the immune response, as also suggested by Dan et al. (2021), who reported 88% persistence for IgG anti-N and 93% for IgG anti-S up to eight months [30].

At the clinical level, differences were observed between symptomatic and asymptomatic individuals. At day 7, IgG anti-N levels were significantly higher among symptomatic patients, and by month 4, IgG anti-S levels also showed significant differences. These findings reinforce existing evidence that symptomatic infection elicits a higher viral loads and more intense immune activation compared to asymptomatic infection [31]. Conversely, no significant associations were found between IgM response and patient sex, comorbidities, or smoking history, consistent with previous studies from Europe and Asia [32]. The absence of such associations may indicate that short-term humoral responses are primarily driven by viral exposure rather than host-related risk factors. This study focused on asymptomatic or mildly symptomatic SARS-CoV-2–infected individuals. Therefore, the interpretation of our findings should be made with caution, as immune responses in these patients may differ substantially from those observed in severe COVID-19 cases.

### Strength and limitations

Strengths: This is the first study in Tunisia to characterize the natural humoral immune response to the original SARS-CoV-2 variant, conducted before the introduction of vaccines or reinfections that could confound the results. It relied on a unique national cohort placed in mandatory isolation with systematic follow-up, which minimized reinfection risk and ensured homogeneous monitoring. The study provided a longitudinal assessment of antibody kinetics (IgM and IgG anti-S/anti-N) over six months, with correlations to clinical presentation. Finally, it contributes original data from a North African population, adding regional and ethnic diversity to the global literature.

Limitations: Patients were enrolled at different times, making it difficult to follow the full serological kinetics of the same cohort across six months and limiting the ability to detect individual variations in immune response. In addition, financial constraints prevented ELISA testing of all collected samples, which reduced the completeness and consistency of data collection and analysis.

## Conclusion

This study described the humoral immune response to the first SARS-CoV-2 variant in a Tunisian cohort, with early IgM and IgG seroconversion, durable IgG persistence up to six months, and higher antibody responses in mild symptomatic cases. These results highlight the ability of the immune system to generate sustained protection, pointing to inter-individual variability influenced by symptom severity. To our knowledge, this is the first national study from Tunisia documenting serological kinetics during the initial COVID-19 wave, thereby contributing valuable data from North Africa to the global understanding of SARS-CoV-2 immunity.
